# Modeling tuberculous meningitis in zebrafish using *Mycobacterium marinum*

**DOI:** 10.1242/dmm.015453

**Published:** 2014-07-04

**Authors:** Lisanne M. van Leeuwen, Martijn van der Kuip, Sameh A. Youssef, Alain de Bruin, Wilbert Bitter, A. Marceline van Furth, Astrid M. van der Sar

**Affiliations:** 1 Department of Pediatric Infectious Diseases and Immunology, VU University Medical Center, De Boelelaan 1117, 1081 HV, Amsterdam, The Netherlands; 2 Department of Medical Microbiology and Infection Control, VU University Medical Center, Van der Boechorststraat 7, 1081 BT, Amsterdam, The Netherlands; 3 Department of Pathobiology, Utrecht University, Faculty of Veterinary Medicine, Yalelaan 1, 3508 TB, Utrecht, The Netherlands

**Keywords:** Tuberculous meningitis, Tuberculosis, Zebrafish, *Mycobacterium marinum*, Blood-brain barrier, ESX-1 mutant

## Abstract

Tuberculous meningitis (TBM) is one of the most severe extrapulmonary manifestations of tuberculosis, with a high morbidity and mortality. Characteristic pathological features of TBM are Rich foci, i.e. brain- and spinal-cord-specific granulomas formed after hematogenous spread of pulmonary tuberculosis. Little is known about the early pathogenesis of TBM and the role of Rich foci. We have adapted the zebrafish model of *Mycobacterium marinum* infection (zebrafish–*M. marinum* model) to study TBM. First, we analyzed whether TBM occurs in adult zebrafish and showed that intraperitoneal infection resulted in granuloma formation in the meninges in 20% of the cases, with occasional brain parenchyma involvement. In zebrafish embryos, bacterial infiltration and clustering of infected phagocytes was observed after infection at three different inoculation sites: parenchyma, hindbrain ventricle and caudal vein. Infection via the bloodstream resulted in the formation of early granulomas in brain tissue in 70% of the cases. In these zebrafish embryos, infiltrates were located in the proximity of blood vessels. Interestingly, no differences were observed when embryos were infected before or after early formation of the blood-brain barrier (BBB), indicating that bacteria are able to cross this barrier with relatively high efficiency. In agreement with this observation, infected zebrafish larvae also showed infiltration of the brain tissue. Upon infection of embryos with an *M. marinum* ESX-1 mutant, only small clusters and scattered isolated phagocytes with high bacterial loads were present in the brain tissue. In conclusion, our adapted zebrafish–*M. marinum* infection model for studying granuloma formation in the brain will allow for the detailed analysis of both bacterial and host factors involved in TBM. It will help solve longstanding questions on the role of Rich foci and potentially contribute to the development of better diagnostic tools and therapeutics.

## INTRODUCTION

In 1993, the World Health Organization (WHO) declared tuberculosis (TB) a global public health emergency, with an estimated 7- to 8-million cases and 1.3- to 1.6-million TB deaths per year. By 2013, the situation had improved in many areas, but absolute numbers remained virtually unchanged, with an estimated 8.7-million new cases and 1.4-million TB deaths per year (World Health Organization, 2013). Central nervous system (CNS) involvement, most commonly leading to tuberculous meningitis (TBM), is the most severe extra-pulmonary complication of TB and accounts for ~1% of all TB cases (Wolzak et al., 2012). TBM especially occurs in early childhood, with a peak incidence in children younger than 5 years (van Well et al., 2009), and has been reported as the most common form of meningitis diagnosed in children in TB endemic areas (Rock et al., 2008). The increased risk of children to develop meningeal TB is presumably due to immature innate and adaptive immune responses (Sterling et al., 2007; Lewinsohn et al., 2004). This immaturity leads to a relative inability to contain primary infection in the lung, which increases the risk of disseminated disease (Sterling et al., 2007; Principi and Esposito, 2012).

After pulmonary TB, tubercle bacilli can disseminate via the bloodstream and are able to cause infection at distant sites such as cervical lymph nodes (scrofula) or vertebrae (Pott’s disease). Our understanding of the pathogenesis of TBM dates from the work of Rich and McCordock in the 1920s and 1930s. They were the first to describe the theory that TBM arises from a caseating granuloma in the brain instead of a direct consequence of a spread of tubercle bacilli to the meninges (Rich and McCordock, 1933). Other researchers, mainly pathologists, confirmed the Rich focus theory (Blacklock and Griffin, 1935; MacGregor and Green, 1937). However, it is not yet clear exactly what mechanisms lead to the formation of foci in the brain and its surrounding meninges. Different types of granulomatous lesions were described in all parts of the CNS, ranging from small and multiple caseous nodules to large exudative plaques (MacGregor and Green, 1937; Rich and Thomas, 1946). A Rich focus can be latent, which causes the disease to remain enclosed for a long period. Alterations in immune status and growth of the granuloma might lead to rupture and discharge of bacteria into the subarachnoid space and meningitis can occur. The inflammatory response to the discharged bacteria results in inflammatory exudation, mostly of the basal cisterns. This can be followed by cranial nerve palsies, obliterative vasculitis, obstruction of cerebral spinal fluid (CSF) and formation of new granulomas elsewhere in the meninges or brain tissue (Rich and Thomas, 1946; Donald et al., 2005). Nowadays, the discussion about the pathogenesis of TBM development and the role of the Rich focus in pathogenesis still continues. A re-interpretation of the work of Rich and McCordock might be required, because a relationship between miliary TB and TBM cannot be completely excluded (Donald et al., 2005). Knowledge about early pathogenesis of TBM is important in the search for early stage diagnostic tools, new treatment strategies and vaccine development.

**RESOURCE IMPACT**

**Background**

Tuberculous meningitis (TBM), involving the central nervous system (CNS), represents the most severe extra-pulmonary complication of tuberculosis (TB). It is caused by infection with *Mycobacterium tuberculosis* and affects, in particular, children below the age of 5 years. Early and rapid diagnosis of TBM is crucial for a favorable outcome; however, owing to non-specific early symptoms, diagnosis is often delayed. When disease progresses, symptoms become more prominent and include focal neurological signs and convulsions, which might lead, in the final stage, to irreversible neurological damage or even death. A clear neuropathological feature of TBM is the formation of granulomas (i.e. collections of immune cells) in the brain tissue or meninges. These pathological structures are the so-called Rich foci, which form after the spread of pulmonary TB via the bloodstream. Little is known about the mechanisms of Rich foci formation and their role in the early pathogenesis of TBM. Therefore, it is necessary to improve knowledge of the initial stages of meningitis development, which might contribute to the design of early stage diagnostic tools as well as of novel therapeutic approaches and vaccines.

**Results**

In this study, the authors adapted the zebrafish model of *M. marinum* infection (zebrafish–*M. marinum* model) to investigate in more detail the early pathogenesis of TBM. They first confirmed that *M. marinum*, a close relative of *Mycobacterium tuberculosis*, could successfully infect adult zebrafish, which have a fully developed immune system and blood-brain barrier, and that there was granuloma formation in the meninges in a minority of cases (as is the case in humans in endemic areas). Subsequently, they characterized the initial phases of zebrafish infection by using different routes of bacteria inoculation at early developmental stages; in most of the zebrafish embryos (which only have innate immunity) both local and systemic injections caused abundant brain infection, with formation of bacterial clusters that were identified as early granulomas. All clusters contained both mycobacteria and a population of either epithelioid or foamy macrophages, but their development was not influenced by the presence of the blood-brain barrier. By contrast, infection with the mycobacteria mutant lacking the ESX-1 secretion system (which is involved in virulence factor secretion) resulted in the formation of smaller clusters with a high number of phagocytosed bacteria, but did not seem to affect migration of bacteria from the bloodstream to the brain tissue.

**Implications and future directions**

This study shows that the zebrafish–*M. marinum* model is particularly suitable for characterization of the early steps in the formation of brain granulomas, their immunological composition and the effect of bacterial virulence factors in the context of TBM. This knowledge is of fundamental importance, particularly considering that the CNS is poorly investigated in the field of tuberculosis research. The advantages of the zebrafish model include its small size, the ease of breeding and of genetic manipulation, as well as the great similarities with the human immune system and blood-brain barrier. Moreover, a unique feature of this model is the transparency of the zebrafish embryos, which, in combination with fluorescent tools, could allow real-time imaging of host-pathogen interactions in the study of infectious diseases, including TBM. This research approach could potentially extend our knowledge of bacterial virulence factors and host characteristics, which will help to advance both early diagnosis and treatment of disease.

Animal experiments have contributed to our knowledge of TB and TBM. A wide range of models to study TB and TBM exists, all with their own benefits and disadvantages. Mice provide a good model to study fundamental features of the immune response to TB and TBM and are used by different research groups (van Well et al., 2007; Be et al., 2008; Zucchi et al., 2012), but a major disadvantage of this model is the fact that mice form poorly organized granulomas after infection with mycobacteria. Animals that do form well-structured granulomas with caseous necrosis are guinea pigs (Rich and McCordock, 1933; Be et al., 2011), rabbits (Tsenova et al., 1998; Tsenova et al., 1999; Tsenova et al., 2002; Tsenova et al., 2006) and non-human primates (Young, 2009). The major disadvantages of these models are the high costs, ethical problems and the limited range of immunology reagents (Young, 2009).

The zebrafish (*Danio rerio*) is a small teleost fish with an innate and adaptive immune system (Lam et al., 2004; Meijer and Spaink, 2011; Renshaw and Trede, 2012) and a blood-brain barrier (BBB), all of which are comparable to humans in both structure and function (Jeong et al., 2008; Xie et al., 2010; Fleming et al., 2013). The first features of the BBB, an endothelial cell layer connected by tight junctions that forms the barrier between the blood and brain tissue (Abbott et al., 2006), are described to be present at 3 days post-fertilization (dpf) (Jeong et al., 2008; Xie et al., 2010), and maturation continues until 10 dpf (Fleming et al., 2013). Zebrafish are extensively used as a model organism; advantages include the fecundation and growth outside the mother, potential to study right from the single-cell embryonic stage, ease of genetic manipulation (Nasevicius and Ekker, 2000) and the availability of a growing mutant library (http://www.sanger.ac.uk/Projects/D_rerio/zmp/). The transparency of zebrafish larvae in combination with an increasing accessibility of fluorescent tools (Ellett et al., 2011; Lawson and Weinstein, 2002; Meijer and Spaink, 2011; Tobin et al., 2012b) provides opportunities to study host-pathogen interaction in real time.

*Mycobacterium marinum* is one of the closest relatives of members of the *Mycobacterium tuberculosis* complex and zebrafish infected with this bacterium develop granulomas similar to those in human TB (Tobin and Ramakrishnan, 2008). It has been shown that mycobacterium-macrophage interaction can initiate granuloma formation in the zebrafish (Davis et al., 2002; Ramakrishnan, 2013). Furthermore, *M. marinum* and *M. tuberculosis* share a lot of important virulence factors, of which the ESX-1 locus is one of the best examples. The mycobacterium ESX-1 locus, encoding a type VII secretion system, plays a role in virulence (Abdallah et al., 2007; Simeone et al., 2012; Stoop et al., 2012) and is partially missing in the vaccine strain *Mycobacterium bovis* Bacillus Calmette-Guérin (BCG) (Gordon et al., 1999). Virulent mycobacteria use the ESX-1 locus to enhance macrophage recruitment and subsequent dissemination of disease (Volkman et al., 2004; Davis and Ramakrishnan, 2009; Ramakrishnan, 2013). The ESX-1 system is required for phagosomal escape (van der Wel et al., 2007; Houben et al., 2012; Simeone et al., 2012), which precedes host cell death. As such, infections with this mutant result in reduced cell death, a reduced number of extracellular bacteria and an increased number of bacteria per infected host cell (Gao et al., 2004).

In this study we set out to determine the optimal inoculation route [intraperitoneally (i.p.) versus intravenous (i.v.) versus direct CNS injection] to induce TBM at different maturation stages of the zebrafish. We show that the zebrafish model of *M. marinum* infection (zebrafish–*M. marinum* model) is an accessible and reproducible model to analyze the pathogenesis of early CNS granuloma formation and the factors involved in this process. In addition, we show that the formation of the BBB does not influence early CNS granuloma formation.

## RESULTS

### I.p. infected adult zebrafish develop granulomas in meninges with brain involvement

**Fig. 1.**
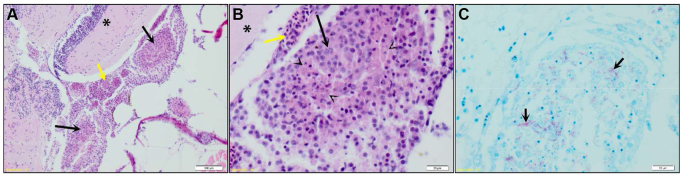
**Granulomas in adult zebrafish after intraperitoneal infection.** (A) Coronal section of adult zebrafish at 50 days after infection with *M. marinum* E11. Multifocal to coalescing granulomas (black arrows) affecting the meninges and submeningeal space structure can be seen, the brain parenchymal tissue (*) beneath the granulomas shows minimal lymphocytic inflammation, and the regional meningeal blood vessels are markedly congested (yellow arrow). Scale bar: 100 μm. Granuloma is enlarged in panels B and C. (B) Granulomas are composed of epithelioid and foamy macrophages (black arrow) that occasionally exhibited degeneration and necrosis (arrowheads). Scale bar: 20 μm. (C) Same granuloma as shown in panel B stained with ZN. Multiple acid-fast bacilli are present in the cytoplasm of the macrophages (black arrows). Scale bar: 20 μm.

To evaluate whether zebrafish can be used to study TBM we first reexamined sections of adult zebrafish acquired in previous experiments (Appelmelk et al., 2008; Stoop et al., 2013) and looked for the presence of mycobacterial infections in the head regions, especially the brain parenchyma or the meninges. These zebrafish were i.p. infected with *M. marinum* at 1 year of age, sacrificed at 8 weeks (±6 days) post infection (wpi) and all fish showed granuloma formation in the abdominal organs. In five out of 26 fish, formation of granulomas (range of one to six granulomas per zebrafish) was also found in close relationship with the brain and meninges. Granulomas mostly affected the meninges and submeningeal space, were multifocal to coalescing, variably sized (50–300 μm in diameter) and fairly circumscribed (Fig. 1A). The brain parenchymal tissue beneath the meningeal granulomas showed minimal lymphocytic inflammation and gliosis, but clear infection of the parenchyma was not observed. The granulomas were composed of a uniform population of epithelioid and foamy macrophages rarely accompanied by the presence of lymphocytes (Fig. 1B, black arrow). Although no clear caseation, calcification or fibrosis was observed, individual macrophages at the center of granulomas exhibited cellular degeneration and necrosis as a first sign of granuloma maturation (Fig. 1B, arrowheads). Ziehl-Neelsen (ZN) staining confirmed the presence of mycobacteria in the cytoplasm of macrophages (Fig. 1C). Interestingly, severe congestion of the meningeal and brain parenchymal blood vessels was present around granulomas. (Fig. 1A,B, yellow arrow). Furthermore, two fish had orbital granuloma formation (supplementary material Fig. S1). Because orbital TB in humans is either a result of direct extension from a tubercular focus in the paranasal sinuses or by hematogenous spread from a distant granuloma, it should not be considered to be the same as CNS TB (Madge et al., 2008). To summarize, in the presence of a full-grown immune system, zebrafish develop granulomas in close relation with brain tissue and meninges after i.p. infection with *M. marinum*. Thereby, we demonstrate that the zebrafish–*M. marinum* infection model is a representative and natural model to study TBM pathogenesis. Interestingly, only a minority of adult fish present infection in the CNS. This is comparable to the human situation in TB endemic areas, where TBM is rare in adults as compared with children (Rock et al., 2008; van Well et al., 2009).

### Zebrafish embryos show abundant early granuloma formation in the brain tissue

#### Local infection

**Fig. 2.**
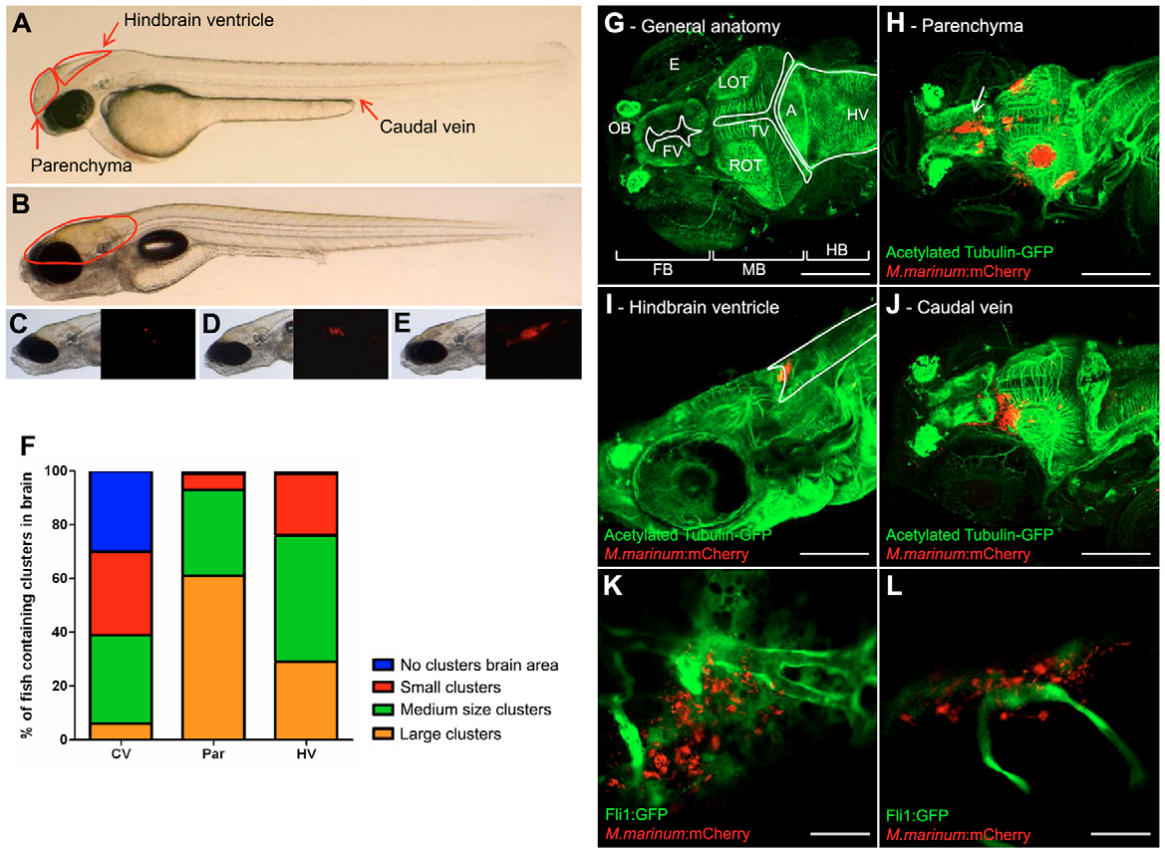
**Three infection routes compared in the embryo model.** (A) Photograph of a casper embryo at 2 dpf. Red arrows indicate the three different infection routes used. (B) Casper embryo at 7 dpf. The red line indicates the brain area. (C) Example of a small cluster. Left panel shows bright-field image; right panel shows corresponding fluorescent image. (D) Example of a medium cluster. (E) Example of a large cluster. (F) At 5 dpi, the infection was analyzed with fluorescence microscopy. Fluorescent bacterial clusters in the brain were counted visually. The clusters were scored, depending on their size, as small, medium or large. CV, caudal vein; Par, parenchyma; HV, hindbrain ventricle. (G–J) Z-stack of zebrafish embryos, at 7 dpf, stained with anti-acetylated tubulin (labelling axons; green) and infected with *M. marinum* E11 (red). Scale bars: 150 μm. (G) Anatomy of the zebrafish brain: FB, forebrain; MB, midbrain; HB, hindbrain; OT, optic tectum (L, left; R, right); OB, olfactory bulb; FV, forebrain ventricle; TV, tectal ventricle; HV, hindbrain ventricle; A, aquaductus; E, eye. (H) Embryo with multiple clusters after infection into the parenchyma. Arrow indicates a cluster in the ventricular wall. (I) Embryo infected into the hindbrain ventricle, with a cluster in the ventricle. Marked area indicates the hindbrain ventricle. (J) Embryo infected via the caudal vein, with a cluster in the parenchyma. (K,L) *Tg(Fli1:GFP)^y1^* casper embryo (Fli1:GFP in green) at 5 days after infection via the caudal vein with *M. marinum* E11 (red). Both panels are single Z-slices and show the relationship between mycobacteria and blood vessels in the brain, which indicates migration of mycobacteria from bloodstream to brain tissue. Scale bars: 35 μm.

To study the effect of *M. marinum* infection in brain tissue, we directly inoculated *M. marinum* into the brain parenchyma or hindbrain ventricle of zebrafish embryos (Fig. 2A; supplementary material Table S1). In 95–100% of all cases this resulted in the formation of bacterial clusters in the targeted brain area (Fig. 2B). Depending on the amount of infection in the brain, infected zebrafish were divided into three groups. Zebrafish were labeled as having large clusters when 50% or more of the brain area contained red fluorescent bacteria, medium clusters when 10–50% was infected and small clusters when less than 10% was infected (Fig. 2C–E). After direct parenchymal infection, large clusters were found in 60% of the cases and only a minority of zebrafish contained small clusters. The clusters formed after hindbrain ventricle infection were mostly medium sized, but large and small clusters were detected as well (Fig. 2F). To obtain more precise information about the localization of these early granulomas, the embryos were histologically analyzed using anti-acetylated tubulin staining of the nerve tracts (Fig. 2G–I; supplementary material Table S1). All infected embryos showed early granuloma formation at the injection site as well as disseminated disease, defined by single bacteria and early granulomas at a distant location (in both parenchyma and ventricular system). Expansion of the primary cluster after parenchymal infection usually took place in the direction of the parenchyma. Interestingly, the bacteria seemed to spread more easily via the cerebral spinal fluid (CSF) in the ventricular system, as exemplified by clusters in the ventricle wall (Fig. 2H, arrow). Similarly, clusters in the ventricle wall were found after hindbrain ventricle infection and displayed clear growth into the parenchyma, indicating that dissemination of bacteria can occur throughout the entire brain after local inoculation of bacteria into the parenchyma and the hindbrain ventricle.

#### Systemic infection

A more natural route for infection of brain tissue is probably via the blood circulation. Therefore, we utilized caudal vein injection as model for hematogenous spread of disease. All 135 examined embryos contained disseminated infection and, of these, 70% (94/135) displayed an infection in the brain area. The amount of embryos with infection in brain tissue seemed to be associated with the number of colony forming units (CFU) used for the systemic injection, i.e. the percentage of zebrafish embryos with brain infection was the lowest (41%) in the experiment with the lowest number of CFU (117 CFU) injected in the caudal vein. In contrast to local brain infection, i.v. infection resulted predominantly in small and medium bacterial clusters in the brain area, whereas large clusters were less commonly seen (Fig. 2F).

**Fig. 3.**
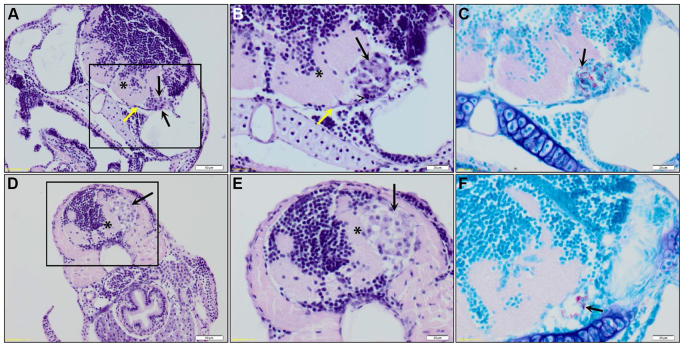
**Histopathological analysis of granulomas in the brain of a zebrafish embryo.** (A) Coronal section of an embryo (7 dpf; 5 days after infection with *M. marinum* E11) showing an early granuloma (black arrows) attached to the meninges (yellow arrow), causing impingement of the underlying brain parenchyma (*). Scale bar: 50 μm. Marked area is enlarged in panels B and C. (B) The early granuloma is composed of epithelioid macrophages (black arrow) and heterophils (arrowhead). (C) ZN stain of granuloma depicted in panel B showing the presence of acid-fast bacilli (black arrow). Scale bars: 20 μm. (D) Coronal section of an embryo (7 dpf; 5 days after infection with *M. marinum* E11) showing an early granuloma (black arrow) present in the submeningeal space causing severe impingement of the underlying brain parenchyma (*). Scale bar: 50 μm. Marked area is enlarged in panels E and F. (E) The early granuloma is composed of foamy macrophages (black arrow) and (F) shows moderate numbers of acid-fast bacilli (black arrow). Scale bars: 20 μm.

Anti-acetylated tubulin staining revealed that the clusters were indeed formed in the brain parenchyma or ventricular system of the zebrafish embryo (Fig. 2J; supplementary material Table S1). Most of the embryos contained more than one cluster and clusters were found to form in every part of the brain. Histopathological analysis [hematoxylin and eosin (HE) staining] confirmed this finding, and showed that meningeal granulomas occasionally impinged on the underlying brain parenchyma causing brain compression and severe brain tissue loss (Fig. 3). To examine the relation between cluster formation and vascular patterns in more detail, *Tg(Fli1:GFP)^y1^* casper zebrafish embryos were used. Infection of these embryos showed that both clustered and single mycobacteria were found in the parenchyma, often closely located to blood vessels (Fig. 2K,L; supplementary material Table S1), indicating that mycobacteria migrate out of blood vessels and form clusters in the brain tissue.

#### Granuloma composition

**Fig. 4.**
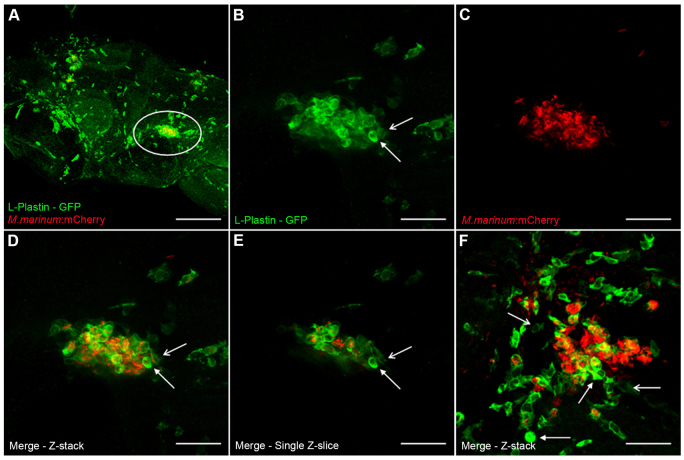
**Composition of early granulomas.** (A) Z-stack of the head of a zebrafish embryo (7 dpf) stained with anti-L-plastin, labeling phagocytic cells (green), infected with *M. marinum* E11 (red) via the caudal vein (5 dpi). Marked area is enlarged in panels B–E. (B–D) Z-stack of an early granuloma, with phagocytic cells stained with anti-L-plastin (B), *M. marinum* E11::mCherry (C) and merge (D). A phagocyte with high L-plastin intensity (closed arrow) and a phagocyte with low L-plastin intensity (open arrow) are depicted in panels B and D. (E) Single Z-slices of granuloma shows colocalization of phagocytes and mycobacteria. Arrows indicate different intensities of L-plastin, eliminating the possible effect of overlapping phagocytes in the Z-stack in panel B and D. (F) Z-stack of an early granuloma containing relatively more mycobacteria than phagocytes. Lots of phagocytes surrounding the cluster are probably still migrating to the cluster. The parenchymal infection route was used. Images were taken at 5 dpi. Scale bars: 150 μm (A), 35 μm (B–F).

To confirm whether the observed bacterial clusters were actually early granulomas containing phagocytic cells, an anti-L-plastin staining was performed. All infection routes showed formation of clusters in which mycobacteria and phagocytes colocalized (Fig. 4; supplementary material Table S2). The phagocyte-mycobacterium area ratio (in confocal images) was equal in ~80% of the observed clusters (supplementary material Table S2). In the remaining clusters, the area covered by mycobacteria was higher compared with the area covered by phagocytes and these clusters were surrounded by many non-infected phagocytes in most cases (Fig. 4F). Interestingly, differences in intensity of L-plastin were observed between different phagocytes inside and around early granulomas (Fig. 4B,D–F, arrows). In addition to confocal microscopy, a more detailed histopathological analysis (HE and ZN staining) of embryos showed that granulomas were composed of a uniform population of either epithelioid or foamy macrophages (Fig. 3A,B,D,E, black arrows) rarely containing heterophils (Fig. 3B, arrowhead), the zebrafish neutrophil counterpart, and contained many acid-fast bacilli (Fig. 3C,F, black arrow). No necrosis or fibrosis was found, fitting with the idea that these are early granulomas. Besides cluster formation, solitary bacterial spread through the brain was observed in all embryos (based on confocal microscopy). These single bacteria were mostly located near a cluster and observed both inside and outside phagocytes (Fig. 4).

### Presence of the BBB does not influence early granuloma formation in the brain parenchyma

**Fig. 5.**
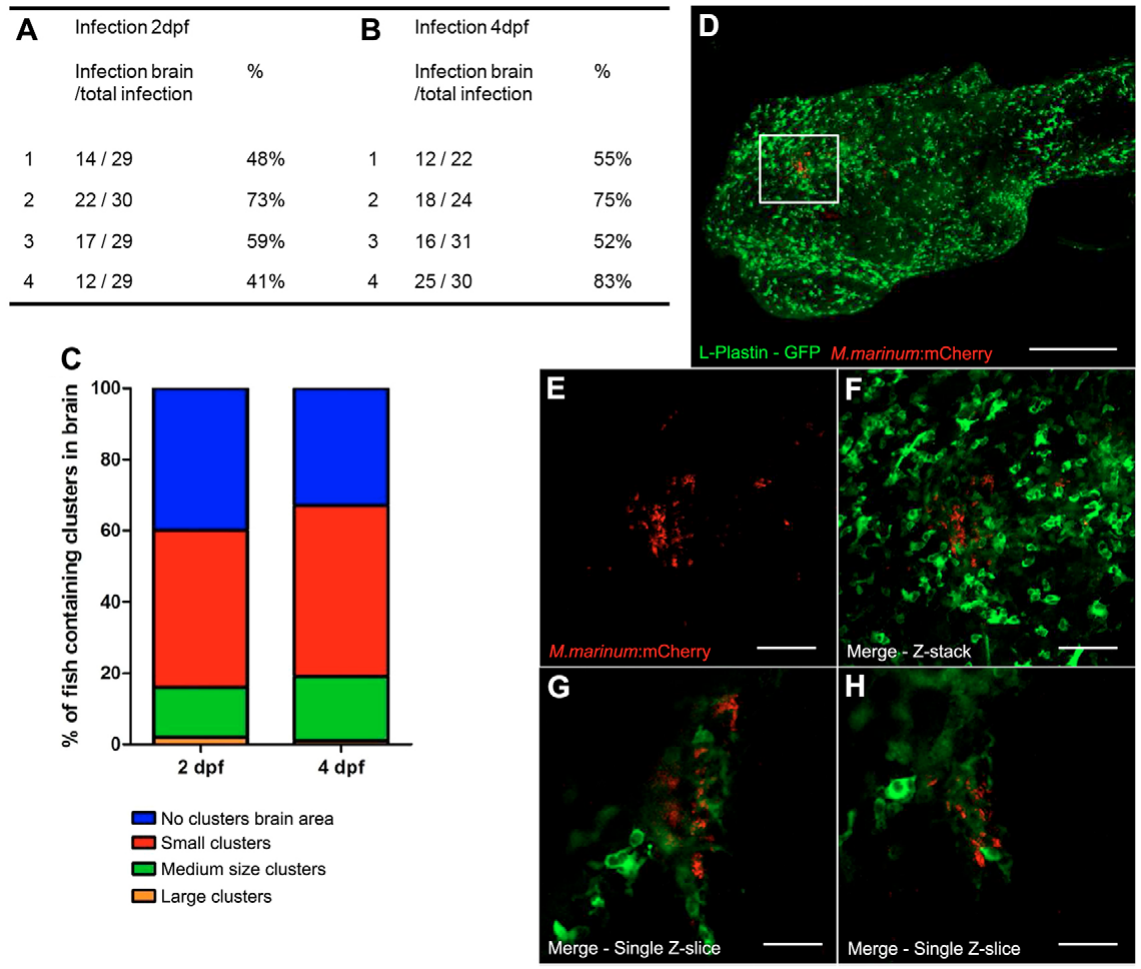
**Presence of the blood-brain barrier.** (A,B) Number of zebrafish embryos with bacteria in the brain area. Embryos were infected either before early BBB development (2 dpf) or after early BBB development (4 dpf) (four groups of embryos were used for each time point). No significant differences were found; *P*=0.3263. (C) Distribution of cluster size is shown as percentages of the total number of infected zebrafish. (D) Casper larvae (32 dpf) microinjected at 21 dpf with *M. marinum* E11 (red) in the heart. Analysis was performed at 11 dpi with anti-L-plastin staining (green). Marked area is enlarged in panels E–H. Scale bar: 300 μm. (E–G) Z-stack of cluster with a lot of phagocytic cells (single channel not shown), mycobacteria (E) and merge of both channels (F). Scale bars: 60 μm. (G,H) Single Z-slices at different Z-levels show colocalization of phagocytes and mycobacteria and cluster formation. Scale bar: 30 μm.

In zebrafish embryos, the BBB is largely formed at 3 dpf, preventing large particles from migrating into the brain parenchyma (Jeong et al., 2008; Xie et al., 2010; Fleming et al., 2013). We confirmed the presence and functionality of this early barrier in our casper fish population (supplementary material Fig. S2) and investigated whether this barrier influences early granuloma formation in the brain. Therefore, embryos were infected i.v. before (2 dpf) and after (4 dpf) the start of BBB formation. The number of zebrafish with infection in the brain, as a percentage of the total number of infected zebrafish, ranged from 41 to 73% for zebrafish infected before BBB development, versus 52–83% after BBB development (Fig. 5A,B). The size of the formed clusters of both groups was equal, i.e. small clusters were mostly observed, medium clusters were less common and large clusters were only seen once or twice (Fig. 5C). These results show that early granulomas can be formed in the brain in the presence of the early BBB and that the presence of this barrier does not seem to influence the amount of infection.

To confirm that even a fully matured BBB does not influence the amount of infection in the CNS after i.v. injection, a group of larvae (21–25 dpf) was infected via the bloodstream. Thirteen out of 71 (18%) infected larvae showed bacterial clusters in the brain area. Interestingly, six of these 13 larvae only showed infection in the brain area. With histochemical analysis using an anti-L-plastin staining and an anti-acetylated tubulin staining, we confirmed that these clusters were actually early granulomas consisting of phagocytic cells and mycobacteria, and were located in the brain parenchyma or ventricles (Fig. 5D–H). The percentage of infection in the larvae brain (21 dpf) was reduced as compared with the embryos (2 or 4 dpf), so further maturation of the BBB might reduce or delay migration of mycobacteria. However, BBB maturation does not seem to block migration, which leads to the assumption that, even in the presence of a fully developed BBB and a partially developed adaptive immune system, bacterial infiltration and cluster formation in the brain occurs efficiently.

### Mycobacteria with a defective ESX-1 secretion system show efficient colonization of brain tissue

**Fig. 6.**
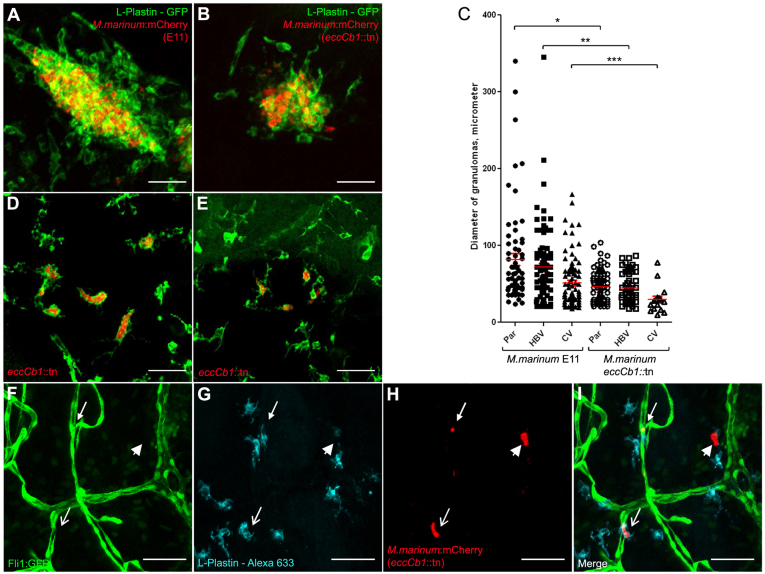
**Role of the ESX-1 locus in the formation of early brain granulomas.** (A) Example of a compact cluster with phagocytic cells and bacteria after infection into the parenchyma with *M. marinum* E11 (red), stained with anti-L-plastin (green). (B) Example of cluster after infection into the parenchyma with *M. marinum eccCb1*::tn (red), stained with anti-L-plastin (green). (C) Overview of diameter of individual granulomas counted in embryos infected via parenchyma (Par), hindbrain ventricle (HBV) or caudal vein (CV), with either *M. marinum* E11 or the isogenic *eccCb1*::tn mutant. The number of granulomas is described in Table 1. Par E11 versus Par F3.1: *P<0.0001, HBV E11 versus HBV F3.1: **P<0.0001, CV E11 versus CV F3.1: ***P=0.0135. (D) Example of single phagocytic cells filled with mycobacteria in an embryo infected into the parenchyma with *eccCb1*::tn mutant (red), stained with anti-L-plastin (green). (E) Example of single phagocytic cells filled with mycobacteria in an embryo infected via the caudal vein with *eccCb1*::tn mutant (red), stained with anti-L-plastin (green). Scale bars: 35 μm (A,B,D,E). (F–I) Z-stack of the relationship between vasculature, phagocytes and bacteria. Caudal vein infection with *M. marinum eccCb1*::tn mutant, with (F) *Tg(Fli1:GFP)^y1^* casper embryo showing blood vessels, (G) L-plastin Alexa-Fluor-633 showing phagocytic cells and (H) *eccCb1*::mcherry showing bacteria. (I) Merge of panels F–H. Closed arrow indicates a phagocyte containing mycobacteria inside a blood vessel; open arrow indicates a phagocyte containing mycobacteria outside a blood vessel; arrowhead shows a bacterial cluster probably inside a dying phagocyte. Scale bars: 50 μm (F–I).

Here, using the *eccCb1*::tn mutant, a bacterial *M. marinum* strain with a disruption in the ESX-1 locus, we examined whether the ESX-1 locus is required for invading the CNS. First, we directly injected the *eccCb1*::tn mutant bacteria into the brain parenchyma or hindbrain ventricle. Similar to what was shown in previous studies (Volkman et al., 2004; Davis and Ramakrishnan, 2009; Stoop et al., 2011), we observed significant differences between the wild-type and the *eccCb1::*tn mutant in cluster formation and cluster size (Table 1; supplementary material Tables S1–S4). In most embryos infected with the *eccCb1*::tn mutant, we only detected small early granulomas (compare Fig. 6A,B). This difference between the early granulomas formed by the *eccCb1*::tn mutant or wild-type mycobacteria is clear upon measuring the diameters (Fig. 6C). Furthermore, in the *eccCb1*::tn mutant group, we observed numerous isolated phagocytes filled with many bacteria in all embryos (Table 1; Fig. 6D). These highly infected phagocytes did not form early granulomas and were scattered throughout the entire brain. In contrast, we never found more than four solitary phagocytes containing mycobacteria in wild-type-infected embryos. Subsequently, we tested the effect on CNS invasion after i.v. infection of the ESX-1 mutant. Again, in these embryos the highly infected extragranulomatous phagocytes seemed to dominate and sporadic early granulomas were only found in half of the embryos (Fig. 6E; supplementary material Tables S3, S4). Importantly, 43% of the *eccCb1*::tn-mutant-injected embryos contained infection in the brain area, indicating that CNS infiltration was not blocked.

**Table 1.**
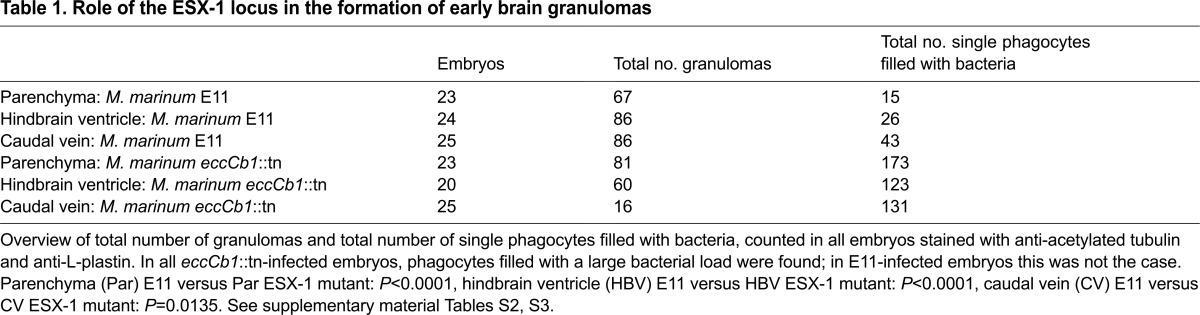
Role of the ESX-1 locus in the formation of early brain granulomas

Closer analysis of *M. marinum eccCb1*::tn-infected *Tg(Fli1:GFP)^y1^* casper embryos, stained with L-plastin, revealed that phagocytes containing mycobacteria were located both inside and outside blood vessels (Fig. 6F–I). This group of ten embryos contained 37 infected phagocytes of which 12 were found inside a blood vessel and 25 were found in the brain tissue (supplementary material Table S2).

In conclusion, infection of zebrafish embryos with the ESX-1 mutant resulted in the formation of smaller clusters and a higher number of phagocytes filled with an abundant load of bacteria, as expected based on earlier publications (Volkman et al., 2004; Davis and Ramakrishnan, 2009). In addition, our experiments indicate that bacterial migration from bloodstream to brain parenchyma still occurs when almost no extracellular bacteria are present.

## DISCUSSION

**Fig. 7.**
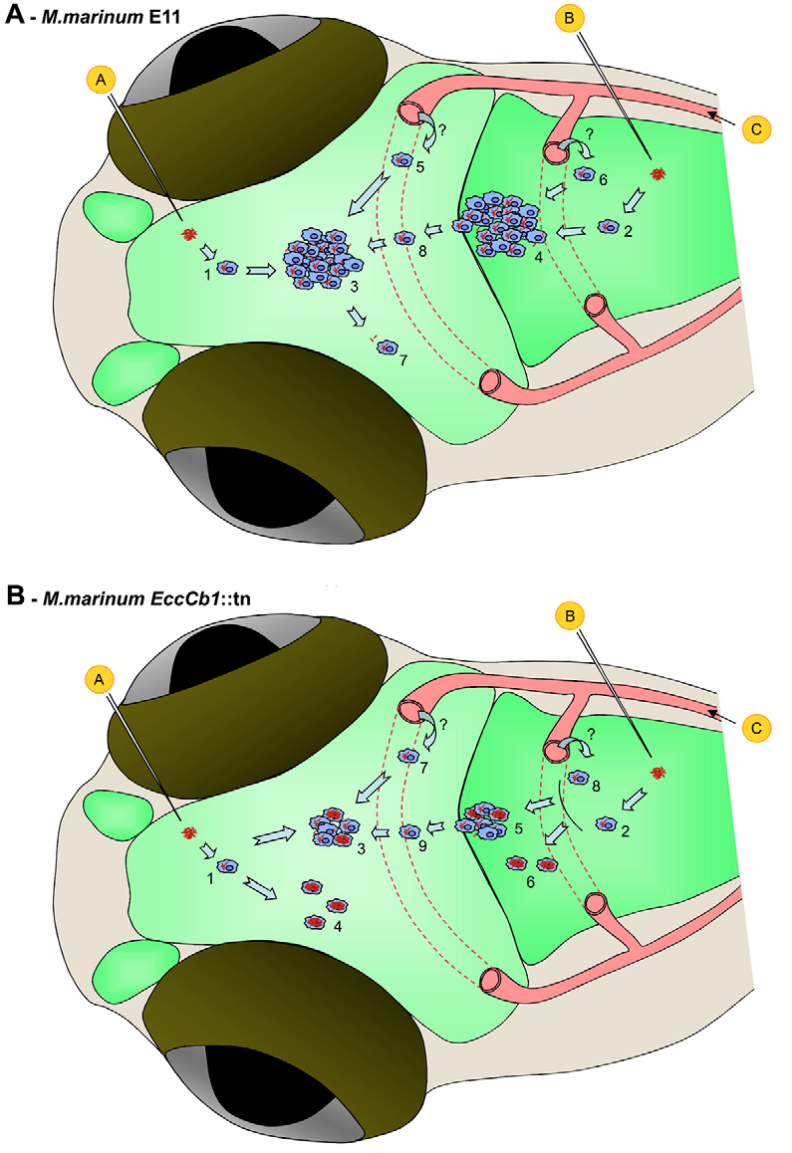
**Graphical abstract: schematic overview of our findings.** (A) Schematic overview of our findings in experiments with *M. marinum* E11. Injection of mycobacteria in the parenchyma (circled ‘A’) or into the hindbrain ventricle (circled ‘B’) leads to uptake by macrophages (1 and 2, respectively) and formation of an early granuloma in the brain parenchyma (3) or the ventricular system (4), respectively. Bloodstream infection (circled ‘C’) also leads to infection of macrophages and cluster formation in the brain parenchyma (5) or the ventricular system (6) by an unknown mechanism (?). Isolated bacteria and infected macrophages can disseminate throughout the brain tissue (7) and can invade the parenchyma via the ventricular system as well (8). (B) Schematic overview of our findings in experiments with *M. marinum EccCb1*::tn. Injection of mycobacteria in the parenchyma (circled ‘A’) or into the hindbrain ventricle (circled ‘B’) leads to uptake by macrophages (1 and 2, respectively) and formation of a small early granuloma in the brain parenchyma (3) or the ventricular system (5), respectively. In addition, isolated highly infected macrophages can be found in the parenchyma (4) or the ventricles (6) in response to injection. Bloodstream infection (circled ‘C’) also leads to infection of macrophages and in some cases to cluster formation in the brain parenchyma (7) or the ventricular system (8) by an unknown mechanism (?). Hypothetically, mycobacteria are transported out of the bloodstream by macrophages in a Trojan-horse mechanism. Isolated bacteria and infected macrophages can invade the parenchyma via the ventricular system as well (9). See supplementary material Table S1–S4.

In this paper we adapted the zebrafish–*M. marinum* infection model to study the early pathogenesis of TBM. From neuropathology studies, it is well known that TBM always starts with granuloma formation in brain tissue or meninges, and our model has the potential to unravel the first steps in the establishment of these granulomas. We show that infection of zebrafish embryos (with innate immunity only) and larvae (with both innate and partial adaptive immunity) via different inoculation routes led to the formation of early granulomas in the brain parenchyma and in the ventricular systems. In addition to the embryo and larval model, granulomas were also observed in the CNS of adult zebrafish after i.p. infection with *M. marinum*. An interpretation of our findings is schematically depicted in Fig. 7.

### Model development

Compared with the adult zebrafish, the embryo and larval TBM model is more flexible and can be tuned depending on the research questions. In zebrafish embryos, differences in cluster size were observed between the three different infection routes. Interestingly, infection via the caudal vein mainly led to the formation of small and medium early granulomas in the brain. Cluster size as well as the number of zebrafish with infections in the brain area seemed to depend on the infection dose, suggesting that severity of TBM correlates with the bacterial load in the blood. The Rich focus theory describes that an early granuloma is formed after hematogenous spread and that TBM is not a direct consequence of miliary TB. In contrast, our results suggest that there might be a direct correlation between higher bacterial load in the blood and higher risk for TBM and higher numbers of granulomas formed in the brain. This is in line with the idea that the Rich focus theory needs to be reconsidered (Donald et al., 2005). An alternative theory is that bacteria spread via the CSF circulation in the ventricular system, which leads to the formation of granulomatous structures or to direct infection of the meninges. Our data shows abundant infection in the ventricular system, which suggests that entrance of mycobacteria into the brain parenchyma via the ventricular wall is a possibility.

### Composition of granulomas in the brain

The major cell types present in early granulomas in embryos and more matured granulomas in adult fish are epithelioid or foamy macrophages. With this histopathological data, it is difficult to determine whether these macrophages have a systemic origin or whether they are derived from microglia (specific brain phagocytes). Interestingly, with the immunohistochemical staining of embryos for the common leukocyte marker L-plastin, we observed differences in L-plastin intensity between the different phagocytes. It is described that macrophages that have colonized the brain and retina undergo a phenotypic transition between 2 and 3 dpf. The expression of L-plastin is downregulated and macrophages start to express high levels of apolipoprotein-E (Herbomel et al., 2001; Meijer and Spaink, 2011). We hypothesize that this downregulation could explain the differences we observed. All granulomas contained phagocytes with high and low L-plastin intensity, which might be an indication that granulomas in the brain consist of microglia and macrophages or neutrophils with a systemic origin. Although we did not find clear caseation of granulomas in brain tissue in our adult fish, signs of maturation, such as the presence of lymphocytes and central necrosis of macrophages, were observed. The similarities in composition between granulomas in embryos and adult fish suggest that embryonal clusters are indeed precursors of adult granulomas. Taking these data together, this model provides opportunities to study the composition and behavior of brain granulomas in an innate versus adaptive setting.

### Bacterial migration

We showed that, after infection via the bloodstream, early granulomas were formed in the brain parenchyma and the ventricular system of both embryos and in larvae containing a fully developed BBB. Our experiments with *Tg(Fli1:GFP)^y1^* casper embryos confirmed these findings and indicated that mycobacteria leave the bloodstream and are able to establish new granulomas in surrounding tissue; because individuals with an intact BBB do develop TBM, it is clear that mycobacteria traverse this barrier. However, the exact mechanism is not yet known.

The BBB consists of endothelial cells with tight junctions, surrounded by a continuous basement membrane and astroglial end-feet (Abbott et al., 2006), which limits the exchange of pathogens, pharmacological compounds, immune cells and mediators (Ransohoff et al., 2003). Three major mechanisms of traversal across this BBB are described for other causative pathogens of meningitis: transcellular migration, paracellular migration or the Trojan-horse mechanism whereby the pathogen uses a macrophage as carrier. *Streptococcus pneumoniae*, *Haemophilus influenzae* and *Neisseria meningitides*, for example, use the transcellular mechanism by binding to the endothelium of the BBB using laminin receptors and by expressing phosphorylcholine, which mimics platelet-activating factor (Orihuela et al., 2009). *Cryptococcus neoformans*, the most common cause of fungal meningitis, has shown to use both the transcellular and the Trojan-horse mechanism.

Liu et al. (Liu et al., 2013) described the dependence of traversal across the BBB on inositol (Liu et al., 2013), of which high concentrations are found in human and animal brains, especially around the BBB. Mycobacteria do have an extensive inositol metabolism, which leads to the hypothesis that host inositol might play an important role in mycobacterial migration over the BBB as well (Morita et al., 2011).

Because mycobacteria are known to be intracellular pathogens, the Trojan-horse mechanism is a plausible hypothesis for traversal across the BBB. *In vitro* studies with a bilayer of alveolar epithelial cells and human lung endothelial cells have strengthened this hypothesis by showing that *M. tuberculosis*-infected monocytes cross the alveolar wall with greater efficiency than uninfected monocytes or mycobacteria alone (Bermudez et al., 2002). Furthermore, invasion of alveolar epithelial cells by mycobacteria enhances this translocation by inducing chemokine release (Bermudez et al., 2002), which suggests that other traversal routes might play an important role as well. The BBB is distinct from the alveolar wall, but the mechanism that mycobacteria and infected macrophages use to cross this barrier might be comparable to what they use in the brain. However, with the current *in vivo* models for TBM, it has never been shown how mycobacteria actually leave the bloodstream and enter the brain. Our adapted zebrafish–*M. marinum* model for TBM is the first in which these mechanisms can be studied.

Zebrafish embryos infected with the ESX-1 mutant contained many individual phagocytes filled with mycobacteria scattered throughout the brain, much like the highly infected individual macrophages scattered throughout the tissue reported by Volkman et al. (Volkman et al., 2004). Interestingly, migration of mycobacteria from the bloodstream to the brain parenchyma and granuloma formation in the brain is therefore not dependent on an intact ESX-1 locus. Attenuated *M. marinum* ESX-1 mutants replicate in the phagolysosome and cannot translocate to the cytosol (van der Wel et al., 2007; Houben et al., 2012). In addition, cell death does not occur as quickly as in wild-type infection and, as a consequence, a lower number of extracellular bacteria are present (Gao et al., 2004; Houben et al., 2012). Together, our results indicate that traversal over the BBB is still possible in a setting with low levels of extracellular bacteria, which leads to the hypothesis that mycobacteria possibly make use of host cells, i.e. macrophages, to migrate out of the bloodstream.

### Clinical implication

Knowledge about the morphological characteristics of granulomas, reflected by systemic and tissue-specific immune responses and mycobacterial virulence factors, is important to improve diagnostic and therapeutic strategies. Histopathological differences in types of granulomas in patients with TBM have been described since the earliest histopathological studies of Rich and McCordock, but also in a recent human postmortem study by our group (D. Zaharie and A.M.v.F., unpublished). In our zebrafish model, we have shown that both embryos and adults can be used to study granuloma composition in great detail, and we observed considerable similarities with human neuropathology. With these two model systems (embryo versus adult), the importance of different granuloma types and influences of innate versus adaptive immunity can be studied in a larger extent. This can subsequently be correlated to inter-individual genetic variability of the innate and/or adaptive immune responses.

A striking finding in our histopathological data of adult zebrafish was the presence of a large amount of congested vessels around meningeal granulomas. In humans, a serious complication of TBM is the development of obliterative vasculitis and subsequent infarction (Rock et al., 2008). Interestingly, this means that this feature of TBM pathogenesis can also be studied in the zebrafish.

In relation to histopathological characteristics of granulomas, susceptibility to infection is another subject of interest. Increased susceptibility to both pulmonary and meningeal TB infection was already shown to be influenced by the Toll-like receptor pathway (Hawn et al., 2006). Recently, inter-individual differences in TNF-α response of zebrafish and humans have been studied (Tobin et al., 2010). TNF-α regulates activation of macrophages, recruitment of other inflammatory cells, induction of cytokine/chemokine production and the induction of apoptosis. Therefore, TNF-α plays a key role in granuloma development. Humans treated with TNF-α neutralizing drugs show increased incidence of TB reactivation (Keane et al., 2001). The protein leukotriene A4 hydrolase, encoded by *LTA4H*, regulates the balance between pro- and anti-inflammatory responses. Individuals with a mutation in both alleles of the *LTA4H* gene develop an anti-inflammatory response with little TNF-α production, whereas individuals having two wild-type alleles have a pro-inflammatory phenotype with abundant TNF-α levels (Tobin et al., 2010; Tobin et al., 2012a). Both scenarios are detrimental to the host, leading to bacterial overgrowth and hyperinflammation, respectively. On the other hand, intermediate TNF-α levels, as seen in individuals who are heterozygous for *LTA4H*, result in moderate inflammation, controlled infection and low risk of severe disease or death (Tobin et al., 2010; Tobin et al., 2012a). It is known that corticosteroids are overall beneficial in TBM outcome (Prasad and Singh, 2008), but thalidomide (a TNF-α inhibitor) seems to work only in a subset of cases (Schoeman et al., 2004; Schoeman et al., 2006). Especially the TB abscess of the CNS is noted to be less responsive to conventional treatment, but good results are reported to anti-TNF therapy in these cases (Schoeman et al., 2006). Thus, from a clinical point of view, knowledge of baseline response levels of TNF-α and subsequent granuloma morphology might have therapeutical consequences in deciding whether to start with inhibiting immunomodulatory drugs, such as corticosteroids or thalidomide. In our model, differences in granuloma number, size and ratio of phagocytes and mycobacteria were observed as well. Also, we showed that mycobacterial virulence factors had a clear impact on granuloma formation and structure. Therefore, this model is an excellent tool to study the formation and behavior of different types of granulomas.

### Conclusion

In conclusion, we have established a reproducible model to study the pathogenesis of TBM in the zebrafish. Our model focused especially on the brain, an organ system that is often neglected. The model can be used for different research questions, and provides opportunities to further extend our knowledge about both bacterial virulence factors that influence granuloma formation and host characteristics leading to differences in type of granuloma and early disease outcome.

## MATERIALS AND METHODS

### Bacterial strains, growth conditions and injection stocks

Two different wild-type strains of *M. marinum* were used in this study: the human isolate, *M. marinum* M strain, originally isolated from human patients with fish tank granulomas (Ramakrishnan and Falkow, 1994), and the sea bass isolate *M. marinum* E11 (van der Sar et al., 2004). In addition, we used the *eccCb1*::tn mutant of E11, which is known to be defective for ESX-1 secretion (Stoop et al., 2011). All *M. marinum* strains were grown at 30°C in Middlebrook 7H9 broth (Difco) with 10% Middlebrook albumin-dextrose-catalase (ADC; BD Bioscience) and 0.05% Tween-80 or on Middlebrook 7H10 agar (Difco) supplemented with 10% oleic-acid-albumin-dextrose-catalase (OADC; BD Bioscience). pSMT3-DsRed and pSMT3-mcherry were electroporated into the *M. marinum* E11 strain, M strain and *eccCb1*::tn (Stoop et al., 2011), in order to be able to visualize bacteria during infection in zebrafish embryos. Transformants of *M. marinum* M strain, E11 and *eccCb1*::tn were selected on plates containing 50 μg/ml hygromycin. Injection stocks were prepared by growing bacteria until the logarithmic phase. Bacteria were washed with 0.3% Tween-80 in phosphate buffered saline (PBS) to declump the bacteria, resuspended in PBS with 20% glycerol and stored at −80°C. Before use, bacteria were resuspended in PBS containing 0.17% (V/V) phenol red (Sigma) to aid visualization of the injection process.

### Animals and injection procedure

Maintenance of adult casper zebrafish (White et al., 2008) and adult *Tg(Fli1:GFP)^y1^* casper zebrafish (Lawson and Weinstein, 2002) took place at 26°C in aerated 5 liter tanks, in a 10:14 hour light:dark cycle. The eggs were collected within the first hour post-fertilization (hpf) and kept at 28°C. At 48 hpf, embryos were mechanically dechorionated and infected by microinjection in the caudal vein, the hindbrain ventricle or the brain parenchyma (Fig. 2A). Injection was performed as described previously (Benard et al., 2012). At 4 days post-infection (dpi) (for *M. marinum* M) or 5 dpi (in the case of *M. marinum* E11 and *M. marinum eccCb1*::tn), bacterial infection was monitored with a Leica MZ16FA fluorescence microscope. Bright-field and fluorescence images were generated with a Leica DC500 (DFC420C) camera and early granuloma formation was analyzed visually and quantified with custom-made software (Stoop et al., 2011) (for additional information, see http://bio-imaging.liacs.nl/galleries/granulomaload/). Larvae were microinjected in the heart at 21–25 dpf, and bacterial infection was monitored at 11 dpi. Following analysis, embryos and larvae were fixed overnight in 4% (V/V) paraformaldehyde (EMS, 100122) dissolved in PBS, and stored in 100% methanol at −20°C for immunohistochemical staining and confocal imaging. To determine the exact number of bacteria injected, the injection volume was also plated on 7H10 plates. During injection and microscopic examining, embryos and larvae were anesthetized in egg water with 0.02% (W/V) buffered 3-aminobenzoic acid (Tricaine; Sigma-Aldrich, A-5040). All procedures involving zebrafish embryos and larvae were performed in compliance with local animal welfare laws. The number of injected embryos and larvae are listed in supplementary material Fig. S3.

### BBB functionality assay with FD4

Fluorescein dextran 4 (FD4, Sigma; 4000 Da) was dissolved in egg water to a final concentration of 2 mg/ml. At 2, 3 and 4 dpf, casper embryos were microinjected with FD4 into the caudal vein. Leakage of FD4 was monitored with confocal microscopy every 10 minutes after injection until 120 minutes post-injection, as described previously (Xie et al., 2010).

### Immunohistochemical stain

After the first screen with fluorescence microscopy, the precise localization and cellular composition of the clusters was determined. Stored infected embryos were stained with anti-L-plastin (Herbomel et al., 1999; Bennett et al., 2001) or anti-acetylated tubulin (Wilson et al., 1990). Anti-L-plastin stains phagocytic cells, whereas anti-acetylated tubulin stains the axonal tracts and commissures of the CNS of the zebrafish (Wilson et al., 1990). With the anatomical atlas developed by the zebrafish workgroup of the University of London (Zebrafish Group and University College London, 2009), we determined important anatomical features that were needed to define the localization of granulomas more precisely (Fig. 2G).

In short, embryos and larvae were rinsed with 1% PBTx, which contains 1% Triton X-100 in PBS, permeated in 0.24% trypsin in PBS and blocked for 3 hours in block buffer, which is 10% normal goat serum (NGS) in 1% PBTx. Incubation with the first antibody was done overnight at room temperature (RT) with anti-L-plastin [1:500 (V/V) dilution] or anti-acetylated tubulin [Sigma T7451, 1:250 (V/V) dilution] in antibody buffer, which is PBTx containing 1% (V/V) NGS and 1% (W/V) BSA. After washing again with PBTx and incubation for 1 hour in block buffer, embryos were incubated in the second antibody overnight at 4°C. For L-plastin staining we incubated in Alexa-Fluor-488 (Invitrogen A11034, 1:200 dilution) or Alexa-Fluor-633 goat-anti-rabbit antibody (Invitrogen A21070, 1:200 dilution); for acetylated tubulin staining we incubated in Alexa-Fluor-488 goat-anti-mouse (Invitrogen A11001, 1:200 dilution). Supplementary material Fig. S3 shows the number of embryos and larvae stained with anti-L-plastin or anti-acetylated tubulin.

### Confocal microscopy

After immunohistochemical staining, embryos were embedded in 1% low-melting-point agarose (Boehringer Mannheim, 12841221-01) dissolved in egg water (60 μg/ml instant ocean see salts) in an 8-well microscopy μ-slide (http://www.ibidi.com). Analysis was performed with a confocal laser scanning microscope (confocal: Leica TCS SP2 with AOBS, microscope: Leica DM IRE2). ImageJ software was used to adjust brightness and contrast and create overlays.

### Histopathological analysis

Histopathological analysis was performed on sections of 1-year-old adult zebrafish, which were i.p. infected with different *M. marinum* strains during experiments performed previously in our laboratory (Appelmelk et al., 2008; Stoop et al., 2013). In addition, we infected casper embryos at 1 dpf with *M. marinum* for histopathological analysis of granulomas in the brain area. Adult zebrafish [8 wpi (±6 days)] and embryos (5 dpi) were anesthetized with MS222, fixed in 4% paraformaldehyde in PBS and horizontally sectioned. Sections of 5 μm were mounted on glass slides and stained with hematoxylin and eosin (HE) or with Ziehl-Neelsen (ZN).

### Graphs and statistical analysis

Graphs in Figs 2, 5 and 6 were made using GraphPad Prism 5.0. Fish were analyzed for each infection route and bars represent mean and standard error of the mean (s.e.m.). Statistical analyses were performed with the same program, using a one-way ANOVA followed by a Bonferroni’s multiple comparison test.

## Supplementary Material

Supplementary Material
