# Supplementary material for: Modeling tuberculous meningitis in zebrafish using Mycobacterium marinum
Source: Dis Model Mech. 2014 Jul 4;7(9):1111–22. doi: 10.1242/dmm.015453 (PMC4142731; doi:10.1242/dmm.015453)
Supplement: Supplementary Material [file supp_7_9_1111__index.html]

Modeling tuberculous meningitis in zebrafish using Mycobacterium marinum — Supplementary Material 

# Modeling tuberculous meningitis in zebrafish using *Mycobacterium marinum*

## DMM015453 Supplementary Material

**Files in this Data Supplement:**

- **Supplementary Material**
