# Supplementary material for: Modeling tuberculous meningitis in zebrafish using Mycobacterium marinum
Source: Dis Model Mech. 2014 Jul 4;7(9):1111–22. doi: 10.1242/dmm.015453 (PMC4142731; doi:10.1242/dmm.015453)
Supplement: Supplementary Material [file supp_7.9.1111_DMM015453.pdf]

SUPPLEMENTARY MATERIALS

Supplementary figure S1: Orbital granulomas

[A] Normal anatomy of orbit and eye of adult zebrafish (NO = nervous opticus, R = retina, OF = orbital fat); [B] Large granulomatous mass (*) in the orbit affecting the optical nerve, after intraperitoneal infection with *M. marinum*; scale bar = 100 µm

Supplementary figure S2: BBB functionality shown with FD4 (4 kDa) as a fluorescent tracer

[A-B] Casper embryo, injected with fluorescent FD4 at 2 dpf via the caudal vein. Distribution of the tracer was monitored with confocal microscopy and shows leakage to the ventricles within the first 30 minutes post injection. [Panel A] Overview of the head of the embryo 90 minutes post injection with accumulation of the tracer in the ventricular system indicating that the BBB is not formed yet at this time point, scale bar = 150 µm, [Panel B] Enlargement of the midbrain ventricle and the hindbrain ventricle with dye accumulation, scale bar = 60 µm. [C-D] Casper embryo, injected with FD4 at 3 dpf via the caudal vein. [Panel C] Overview of the head of the embryo 90 minutes post injection. A clear pattern of the blood vessels is visible with minimal leakage of fluorescent dye to the ventricular system, Scale bar = 150 µm. [Panel D] Enlargement of Panel C, same region as Panel B, Scale bar = 60 µm. [E-F] Casper embryo, injected with FD4 at 4 dpf via the caudal vein. [Panel E] Overview of the head of the embryo 90 minutes post injection. A clear pattern of the blood vessels is visible without leakage of fluorescent dye to the ventricular system, indicating that the BBB is functional at 4 dpf in limiting traversal of molecules of 4 kDa and larger, scale bar = 150 µm. [Panel F] Enlargement of Panel E, same region as Panel B and D, scale bar = 60 µm.

Supplementary figure S3: Method

[A] This diagram shows the injection procedure, the number of infected embryos per injection route and the CFU counts injected, for *M. marinum* E11, *M. marinum ecCb1::tn*, phenol red/PBS as control group and *M. marinum* M. We used casper and *Tg(fli1:GFP)* zebrafish. The number of embryos stained with anti-L-plastin and anti-acetylated tubulin is depicted in the bottom of A.

[B] This diagram shows the procedure followed for experiments with zebrafish larvae. Larvae were infected via the heart at 21-25 dpf, analysis took place 11 dpi. dpf = days post fertilization; dpi = days post infection.

Supplementary table S1

This table shows all casper and *Tg(Fli1:GFP)*^{y1} casper embryos infected with *M. marinum* E11 and stained with anti-acetylated tubulin (in cases of non-transgenic zebrafish) to determine localization of early granulomas.

Supplementary table S2

This table shows all casper embryos infected with *M. marinum* E11 and stained with anti-L-plastin to analyse cluster composition and confirm that these clusters are indeed early granulomas.

Supplementary table S3

This table shows all casper and *Tg(Fli1:GFP)*^{y1} casper embryos infected with *M. marinum eccCb1*::tn and stained with anti-acetylated tubulin (in cases of non-transgenic zebrafish) to determine localization of early granulomas.

Supplementary table S4

This table shows all casper embryos infected with *M. marinum eccCb1*::tn and stained with anti-L-plastin to analyse cluster composition and confirm that these clusters are indeed early granulomas.

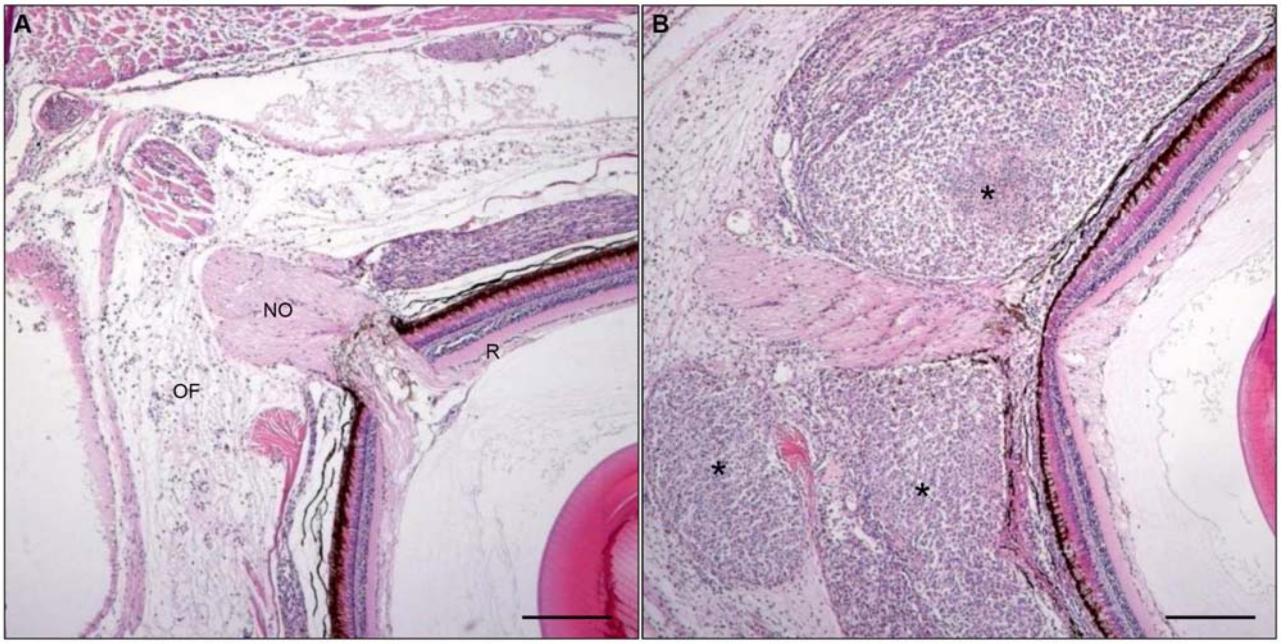

Supplementary figure S1

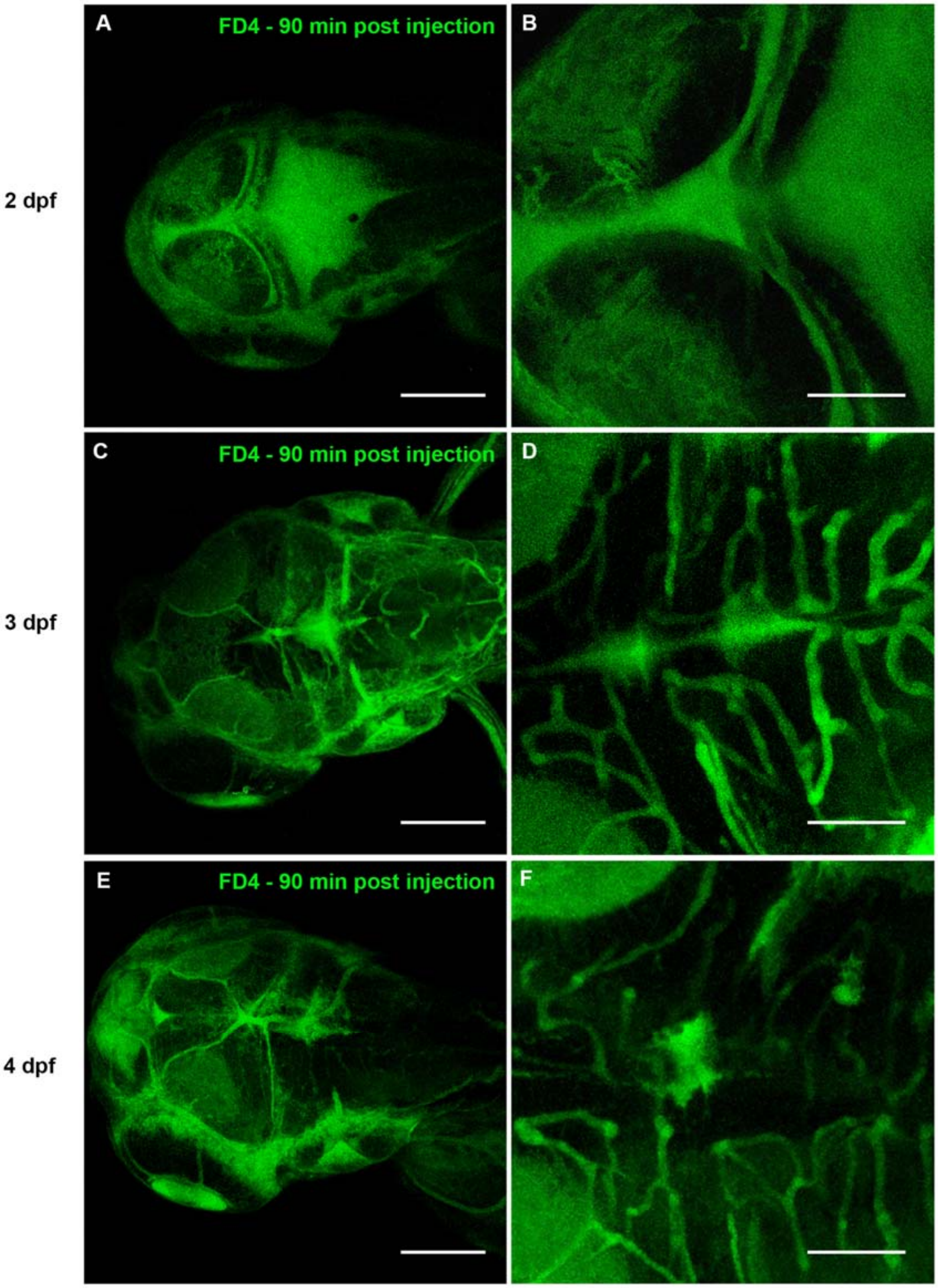

Supplementary figure S2

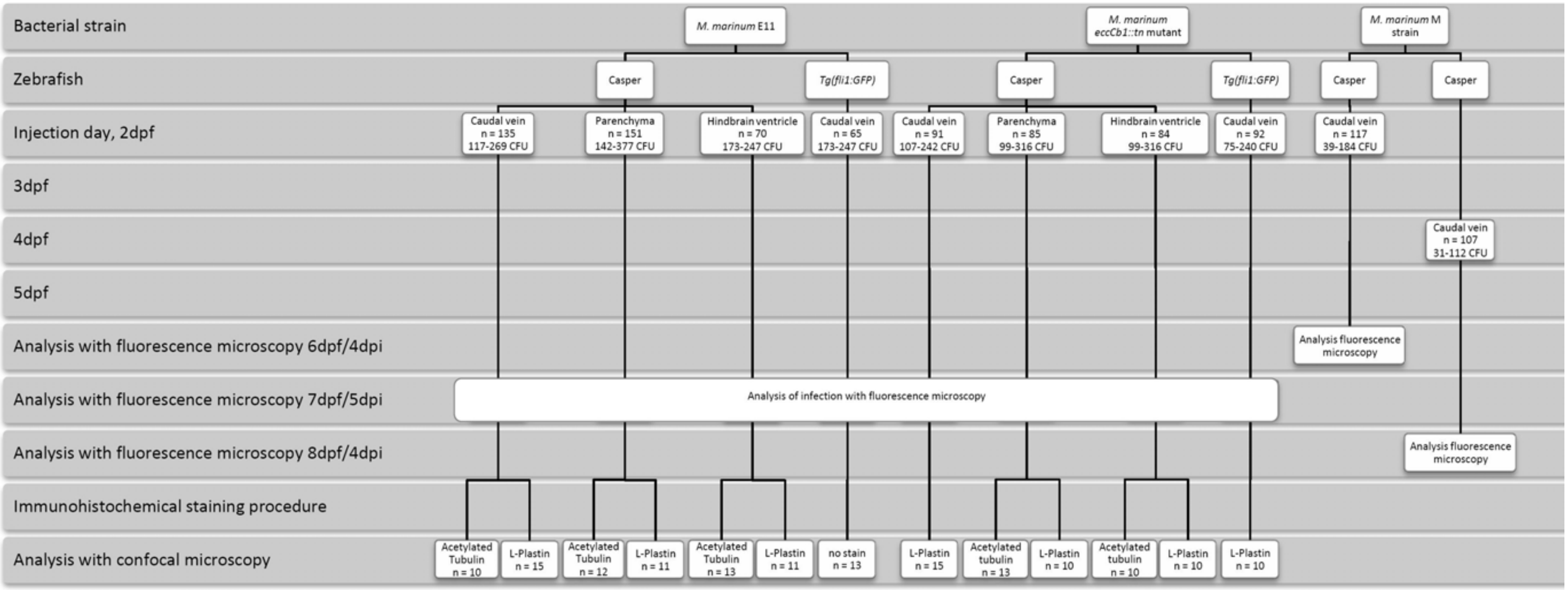

Supplementary Figure 3A

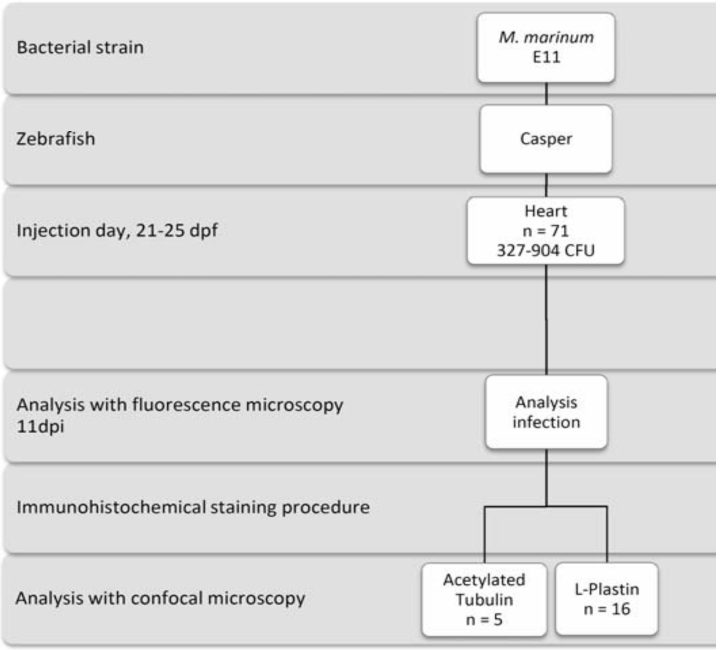

Supplementary Figure 3B

Route of injection		Number
**Parenchyma**	Number of infected zebrafish analyzed with fluorescence microscopy	151 (9 experiments, 8-23 embryos per experiment)
	CFU injected	142-377 CFU (mean: 230 CFU)
	- Bacterial clusters in brain area	95-100%
	Number of zebrafish stained with anti-acetylated tubulin	12
	Total number of early granulomas	36 (1-7/embryo)
	Number of early granulomas at injection spot	11 (11/12 embryos)
	Disseminated disease	10 embryos
	- Early granulomas at distant location	25 (10/12 embryos)
	o Expansion of primary cluster via parenchyma	13/25 (10/10 embryos)
	o Expansion/dissemination via CSF	12/25 (9/10 embryos)
	- Single bacteria	10/12 embryos
	o In parenchyma	9/10 embryos
	o In ventricular system	9/10 embryos
	Early granulomas in ventricular wall	9 (7/12 embryos)
	- Without invasion of parenchyma	2/9
	- With invasion of parenchyma	7/9
**Hindbrain ventricle (HBV)**	Number of infected zebrafish analyzed with fluorescence microscopy	70 (4 experiments, 15-22 embryos per experiment)
	CFU injected	173-377 CFU (mean: 260 CFU)
	- Bacterial clusters in brain area	95-100%
	Number of zebrafish stained with anti-acetylated tubulin	13
	Total number of early granulomas	48 (1-8/embryo)
	Number of early granulomas at injection spot	25/48 (13/13 embryos)
	Disseminated disease	13 embryos
	- Early granulomas at distant location	23/48 (9/13 embryos)
	o Single clusters in parenchyma	9/23 (6/9 embryos)
	o Located in ventricular system (other than HBV)	14/23 (8/9 embryos
	- Single bacteria	13/13 embryos
	o In parenchyma	11/13 embryos
	o In ventricular system	11/13 embryos
	Early granulomas in ventricular wall (HBV included)	12/48 (9/13 embryos)
	- Without invasion of parenchyma	2/12
	- With invasion of parenchyma	10/12
**Caudal Vein**	Number of infected zebrafish analyzed with fluorescent microscopy	135 (5 experiments, 18-44 embryos per experiment)
	CFU injected	117-269 CFU (mean: 197 CFU)
	- Bacterial clusters in brain area	70%
	Number of zebrafish stained with anti-acetylated tubulin	10
	Total number of early granulomas	30 (1-6/embryo)
	Localization of early granulomas	
	- Parenchyma	
	o Hindbrain	6/30
	o Midbrain	16/30
	o Forebrain	3/30
	- Ventricles	
	o Hindbrain ventricle	3/30
	o Midbrain ventricle	2/30
	o Forebrain ventricle	-
	Number of infected *Tg(Fli1:GFP)*^{y1} casper embryo analyzed with fluorescent microscopy	65 (experiments, embryos per experiment)
	CFU injected	173-247 CFU (mean: CFU)
	Number of *Tg(Fli1:GFP)*^{y1} casper embryo analyzed with confocal microscopy	17 (4/17 L-Plastin, 13/17 without additional staining)
	Total number of early granulomas	48 (1-8/embryo)
	- Early granulomas in close relationship with vasculature	18/48 (11 embryos)
	- Early granulomas solitary in parenchyma	30/48 (17 embryos)

Single bacteria	16/17 embryos
- In close relationship with vasculature	13/16
- Solitary in parenchyma	15/16

Supplementary table S1

Route of injection		Number
**Parenchyma**	Number of infected zebrafish analyzed with fluorescence microscopy	151 (9 experiments, 8-23 embryos per experiment)
	CFU injected	142-377 CFU (mean: 230 CFU)
	- Bacterial clusters in brain area	95-100%
	Number of zebrafish stained with anti-L-plastin	11
	Total number of early granulomas	31 (1-5/embryo)
	Single phagocytes filled with a large amount of bacteria	6 (4/11 embryos)
	Ratio bacteria – phagocytes	
	- Bacteria = phagocytes	19/31
	- Bacteria > phagocytes	11/31
	- Bacteria < phagocytes	1/31
**Hindbrain ventricle**	Number of infected zebrafish analyzed with fluorescence microscopy	70 (4 experiments, 15-22 embryos per experiment)
	CFU injected	173-377 CFU (mean: 260)
	- Bacterial clusters in brain area	95-100%
	Number of zebrafish stained with anti-L-plastin	11
	Total number of early granulomas	38 (1-7/embryo)
	Single phagocytes filled with a large amount of bacteria	10 (4/11 embryos)
	Ratio bacteria – phagocytes	
	- Bacteria = phagocytes	33/38
	- Bacteria > phagocytes	5/38
	- Bacteria < phagocytes	0/38
**Caudal Vein**	Number of infected zebrafish analyzed with fluorescence microscopy	135 (5 experiments, 18-44 embryos per experiment)
	CFU injected	117-269 CFU (mean: 197 CFU)
	- Bacterial clusters in brain area	70%
	Number of zebrafish stained with anti-L-plastin	11
	Total number of early granulomas	44 (2-11/embryo)
	Single phagocytes filled with a large amount of bacteria	19 (9/11 embryos)
	Ratio bacteria – phagocytes	
	- Bacteria = phagocytes	38/44
	- Bacteria > phagocytes	6/44
	- Bacteria < phagocytes	0/44

Supplementary table S2

Route of injection		Number
**Parenchyma**	Number of infected zebrafish analyzed with fluorescence microscopy	85 (4 experiments, 18-24 embryos per experiment)
	CFU injected	99-316 CFU (mean: 200 CFU)
	- Bacterial clusters in brain area	96-100%
	Number of zebrafish stained with anti-acetylated tubulin	13
	Total number of early granulomas	44 (1-5/embryo)
	Number of early granulomas at injection spot	10/44 (10/13 embryos)
	Disseminated disease	
	- Early granulomas at distant location	34 (11/13 embryos)
	o Expansion of primary cluster via parenchyma	9 (10/11 embryos)
	o Expansion/dissemination via CSF	25 (11/11 embryos)
	- Single bacteria	13/13 embryos
	o In parenchyma	10/13 embryos
	o In ventricular system	11/13 embryos
	Early granulomas in ventricular wall	17 (11/13 embryos)
	- Without invasion of parenchyma	4/17
	- With invasion of parenchyma	13/17
**Hindbrain ventricle (HBV)**	Number of infected zebrafish analyzed with fluorescence microscopy	84 (4 experiments, 18-23 embryos per experiment)
	CFU injected	99-316 CFU (mean: 200 CFU)
	- Bacterial clusters in brain area	88-100%
	Number of zebrafish stained with anti-acetylated tubulin	10
	Total number of early granulomas	23 (1-5/embryo)
	Number of early granulomas at injection spot	8 (7/10 embryos)
	Disseminated disease	
	- Early granulomas at distant location	15 (8/10 embryos)
	o Single clusters in parenchyma	8 (5/8 embryos)
	o Located in ventricular system (other than HBV)	7 (6/8 embryos)
	- Single bacteria	10/10 embryos
	o In parenchyma	9/10 embryos
	o In ventricular system	10/10 embryos
	Early granulomas in ventricular wall (HBV included)	9 (8/10 embryos)
	- Without invasion of parenchyma	2/9
	- With invasion of parenchyma	7/9
**Caudal Vein**	Number of infected *Tg(Fli1:GFP)*^{y1} casper embryos analyzed with fluorescence microscopy	92 (3 experiments, 29-33 embryos per experiment)
	CFU injected	75-240 CFU (mean:163 CFU)
	- Bacterial clusters in brain area	53%
	Number of *Tg(Fli1:GFP)*^{y1} casper embryos stained with anti-L-plastin analyzed	10
	Total number of early granulomas	8 (1-3/embryo, 5 embryos)
	- Early granulomas in close relationship with vasculature	1/8
	- Early granulomas solitary in parenchyma	7/8
	Total number of single phagocytes filled with a large amount of bacteria	39 (1-8/embryo, 10 embryos)
	- Inside blood vessel	12/39 (5 embryos)
	- Outside blood vessel	27/39 (10 embryos)

Supplementary table S3

Route of injection		Number
**Parenchyma**	Number of infected zebrafish analyzed with fluorescence microscopy	85 (4 experiments, 18-24 embryos per experiment)
	CFU injected	99-316 CFU (mean: 200 CFU)
	- Bacterial clusters in brain area	96-100%
	Number of zebrafish stained with anti-L-plastin	10
	Total number of early granulomas	37 (1-9/ embryo, 10 embryos)
	Single phagocytes filled with a large amount of bacteria	85 (1-19/ embryo, 10 embryos)
	Ratio bacteria – phagocytes, granulomas	
	- Bacteria = phagocytes	30/37
	- Bacteria > phagocytes	3/37
	- Bacteria < phagocytes	4/37
**Hindbrain ventricle**	Number of infected zebrafish analyzed with fluorescence microscopy	84 (4 experiments, 18-23 embryos per experiment)
	CFU injected	99-316 CFU (mean: 200 CFU)
	- Bacterial clusters in brain area	88-100%
	Number of zebrafish stained with anti-L-plastin	10
	Total number of early granulomas	39 (1-7/embryo, 10 embryos)
	Single phagocytes filled with a large amount of bacteria	60 (1-10/embryo, 10 embryos)
	Ratio bacteria – phagocytes	
	- Bacteria = phagocytes	29/39
	- Bacteria > phagocytes	1/39
	- Bacteria < phagocytes	7/39
**Caudal Vein**	Number of infected casper embryos analyzed with fluorescence microscopy	91 (3 experiments, 29-31 embryos per experiment)
	CFU injected	107-242 CFU (mean:165 CFU)
	- Bacterial clusters in brain area	33%
	Number of infected *Tg(Fli1:GFP)*^{y1} casper embryos analyzed with fluorescence microscopy	92 (3 experiments, 29-33 embryos per experiment)
	CFU injected	75-240 CFU (mean:163 CFU)
	- Bacterial clusters in brain area	53 %
	Number of zebrafish stained with anti-L-plastin	25
	Total number of early granulomas	16 (1-3/embryo, 13 embryos)
	Single phagocytes filled with a large amount of bacteria	132 (1-15/embryo, 25 embryos)
	Ratio bacteria – phagocytes	
	- Bacteria = phagocytes	15/16
	- Bacteria > phagocytes	0/16
	- Bacteria < phagocytes	1/16

Supplementary table S4
